# Indirect pulp therapy with selective removal of carious dentin in permanent teeth: An *in vivo* study with one year follows up

**DOI:** 10.6026/973206300211367

**Published:** 2025-06-30

**Authors:** Khushbu Barak, Ashtha Arya, Labishetty Karthik, Aksharkumar J Patel, Vinod Kumar A., Tejaswi Kala

**Affiliations:** 1 Department of Conservative Dentistry and Endodontics; SGT Dental College, Hospital and Research Institute, SGT University, Gurugram, Haryana, India; 2 Department of Conservative Dentistry and Endodontics, Tirumala Institute of Dental Sciences and Research Center, Nizamabad, Telangana, India; 3 Registered Dental Assistant (Level 1 & 2), Scott Family Dental Office, Thunder Bay, Ontario, Canada - P7B1C5; 4 Department of Conservative Dentistry and Endodontics, Meghna Institute of Dental Sciences, Nizamabad, Telangana, India; 5 Department of Public Health Dentistry, Tirumala Institute of Dental Sciences and Research Center, Nizamabad, Telangana, India

**Keywords:** Indirect pulp capping, calcium hydroxide, selective caries removal, tertiary dentin, deep caries

## Abstract

The aim of the present study is to evaluate the efficacy of indirect pulp therapy (IPT) with selective removal of carious dentin in deep carious lesions of permanent teeth. One hundred permanent molars and premolars with deep carious lesions and no signs of irreversible pulpitis were selected. Selective caries removal was performed, preserving a layer of soft dentin over the pulpal wall. Calcium hydroxide liner was applied, followed by composite restoration. Clinical and radiographic follow-ups were conducted at 1, 6 and 12 months. At the 12-month visit, the cavity was re-entered to assess tertiary dentin formation. At 12 months follow up seventy-three cases were successful, with only two failures, resulting in a success rate of 97.3%.

## Background:

Pulp vitality plays a critical role in the long-term preservation and function of the tooth. Conventionally, root canal treatment (RCT) has been the standard intervention following a diagnosis of pulpitis; it involves loss of vital tissue and structural integrity, increasing the risk of root fracture. Vital pulp therapy (VPT) offers a conservative approach, aiming to retain pulp function and arrest disease progression [1]. Indirect pulp therapy (IPT) involves selective removal of carious dentin and placement of a protective liner, such as calcium hydroxide, to promote healing [1]. This study investigates a modified IPT technique where infected dentin is removed from lateral walls, while a thin layer of affected dentin is preserved over the pulp. Uniquely, the study includes clinical re-entry after one year to evaluate tertiary dentin formation. To date, no study has assessed this outcome through direct clinical observation, making this an important contribution to evidence-based VPT strategies.

## Materials and Methods:

This prospective clinical study evaluated the success of minimally invasive caries removal in 100 patients presenting with deep carious lesions and a clinical diagnosis of reversible pulpitis. Preoperative radiographs confirmed the absence of periapical pathology and pulp sensibility tests were positive. After achieving local anaesthesia and rubber dam isolation, caries removal was performed using a slow-speed round bur and spoon excavator. Infected dentin was completely removed from the lateral walls, while a thin layer of affected dentin was intentionally retained over the pulpal wall to prevent pulp exposure. A calcium hydroxide liner was placed, followed by restoration with composite resin. Patients were evaluated at 1, 6 and 12 months postoperatively. Clinical success was determined by the absence of pain, continued positive sensibility responses and no signs of pulp necrosis or periapical pathology. In cases of clinical failure, root canal treatment was performed. At the 12-month follow-up, the cavity was re-entered to directly evaluate the formation of tertiary dentin. The primary outcome was the overall success rate based on pulp vitality, restoration function and evidence of dentin bridge formation.

## Results and Discussion:

No significant differences were observed in most clinical parameters; however, dentin color and consistency showed statistically significant changes. At baseline, 76% of cases had light yellow dentin, while only 3% were dark brown. After 12 months, none remained light yellow and 73.33% exhibited dark brown dentin, indicating a marked color shift over time. Regarding consistency, 86% of participants initially presented with "very soft" dentin, 9% with "soft" and 5% with "medium hard". At the 12-month follow-up (n=75), only 1.33% remained "very soft" or "soft" and 97.33% demonstrated "hard" dentin. Minimally invasive techniques like vital pulp therapy (VPT) are emphasized over conventional root canal therapy (RCT), which carries risks such as root fracture and diminished protective mechanism against masticatory stresses [2]. VPT, including Indirect Pulp Capping (IPC), seeks to preserve pulp vitality, especially when reversible pulpitis is diagnosed [3]. Once caries is excavated if there is no pulpal exposure, pulp tissue still has ability to heal, as dentin contains several hundred matrix-bound proteins, including cytokines, neurotrophic, angiogenic factors and other growth factors [4]. They can be liberated through demineralization and their subsequent release can alter the immune response as well as the dental pulp cells' capacity for migration, proliferation, differentiation and mineralization. Therefore, following indirect pulp therapy, dentin matrix proteins promote healing and repair [2]. The study followed selective soft dentin removal and placement of calcium hydroxide, known for antibacterial properties, remineralization and biocompatibility. This process is known as "stimulating the residual pulpal tissue to facilitate the regeneration of the dental pulp complex" [3]. Tissue near pulp that has become demineralized or infected by bacteria may be left in situ. This kind of management could involve a step-by-step caries excavation process or a two-step process [4]. According to a study by Rathi [5] when re-entering the cavity for the complete eradication of caries in the second appointment, there may be an unnecessary risk of pulp exposure, they recommended single visit indirect pulp capping, in which the entire process is completed in a single visit and the tooth is not revisited again for entire caries removal which is followed in our study [5]. Despite MTA's effectiveness, its discoloration effects led to selection of calcium hydroxide, which yielded a 97.3% success rate over 12 months. Research supports calcium hydroxide as a gold standard for pulp conservation due to antimicrobial and reparative effects. Consistency and color of dentin were assessed, showing a statistically significant change from soft yellow to hard dark brown dentin, suggesting tertiary dentin formation [6]. These results affirm the role of Maillard reactions and bacterial isolation in lesion arrest and hardening [6]. The study's major limitation was the lack of microbiological or histological data. Nevertheless, rigorous protocols involving rubber dam isolation, standardized procedures and biocompatible materials like Dycal and composite strengthened the study's validity [7, 8]. Future *in vivo* studies with extended observation periods are necessary to evaluate the effectiveness of single-visit IPT in permanent teeth.

## Financial support and sponsorship:

Nil.

## Conclusion:

Indirect pulp therapy with calcium hydroxide, involving selective removal of soft dentin, has been shown to effectively preserve pulp vitality and stimulate the formation of tertiary dentin.
